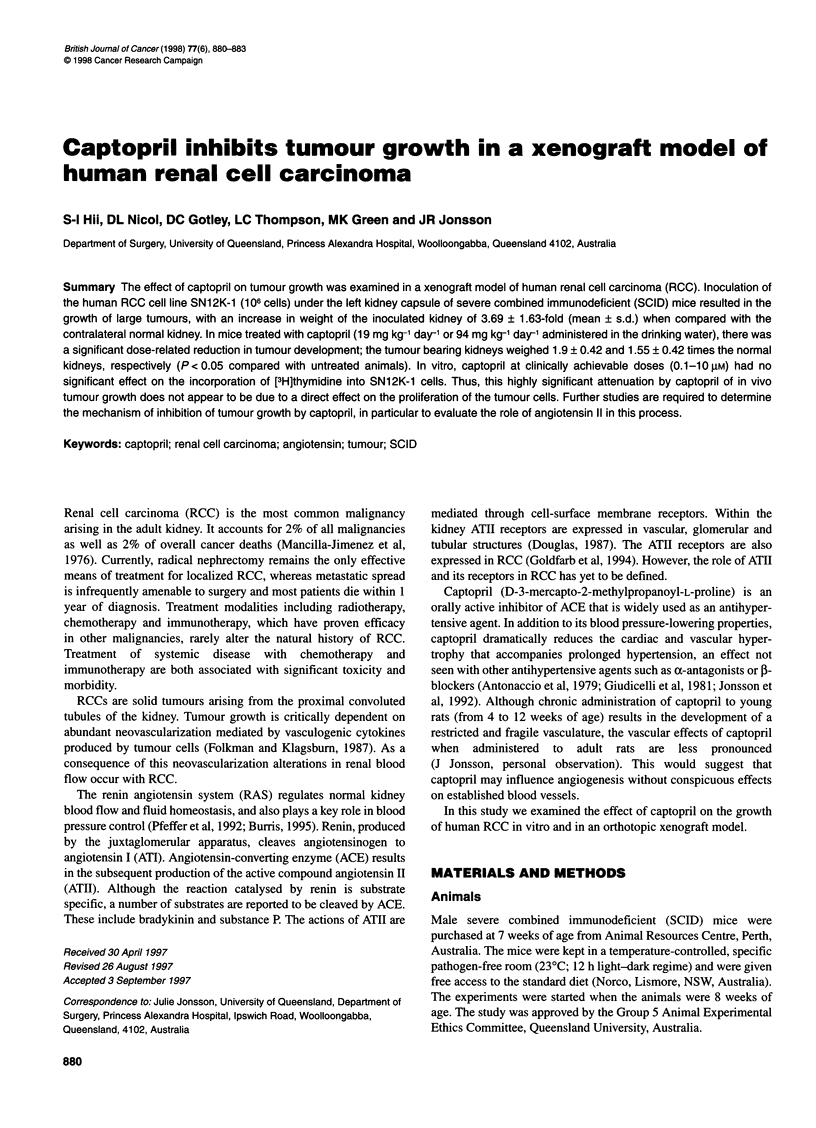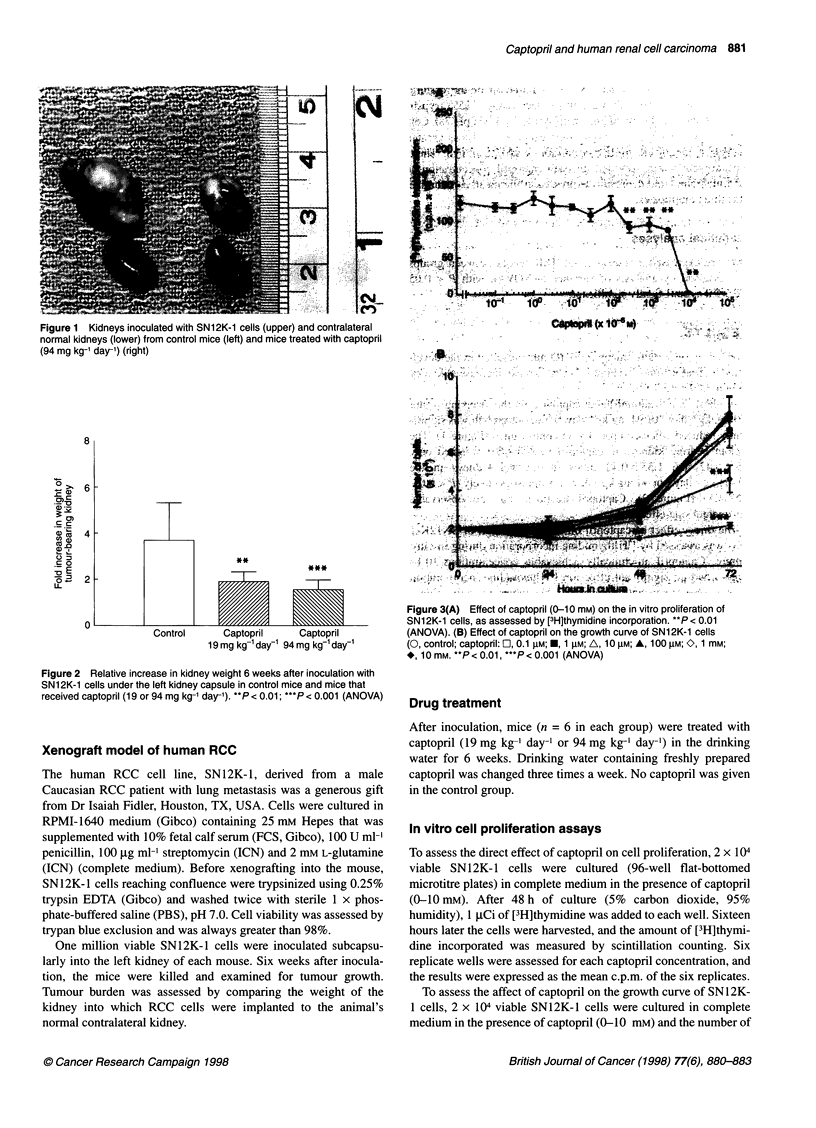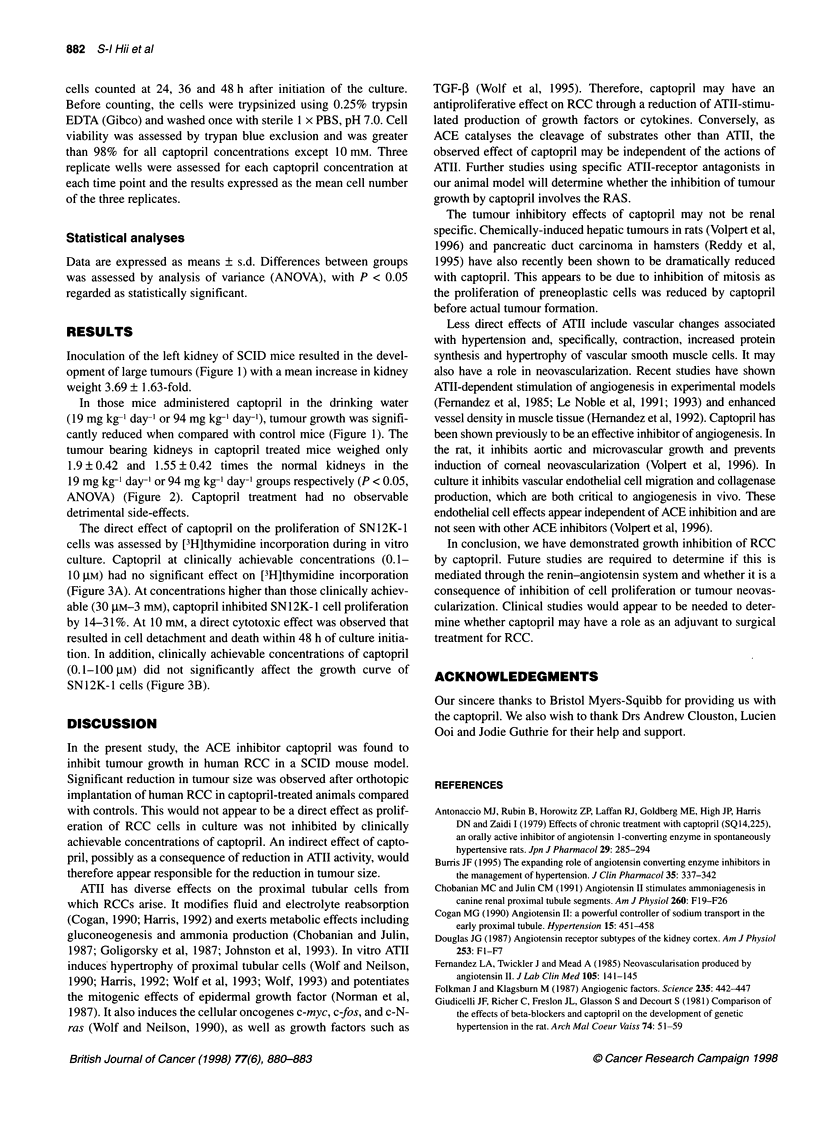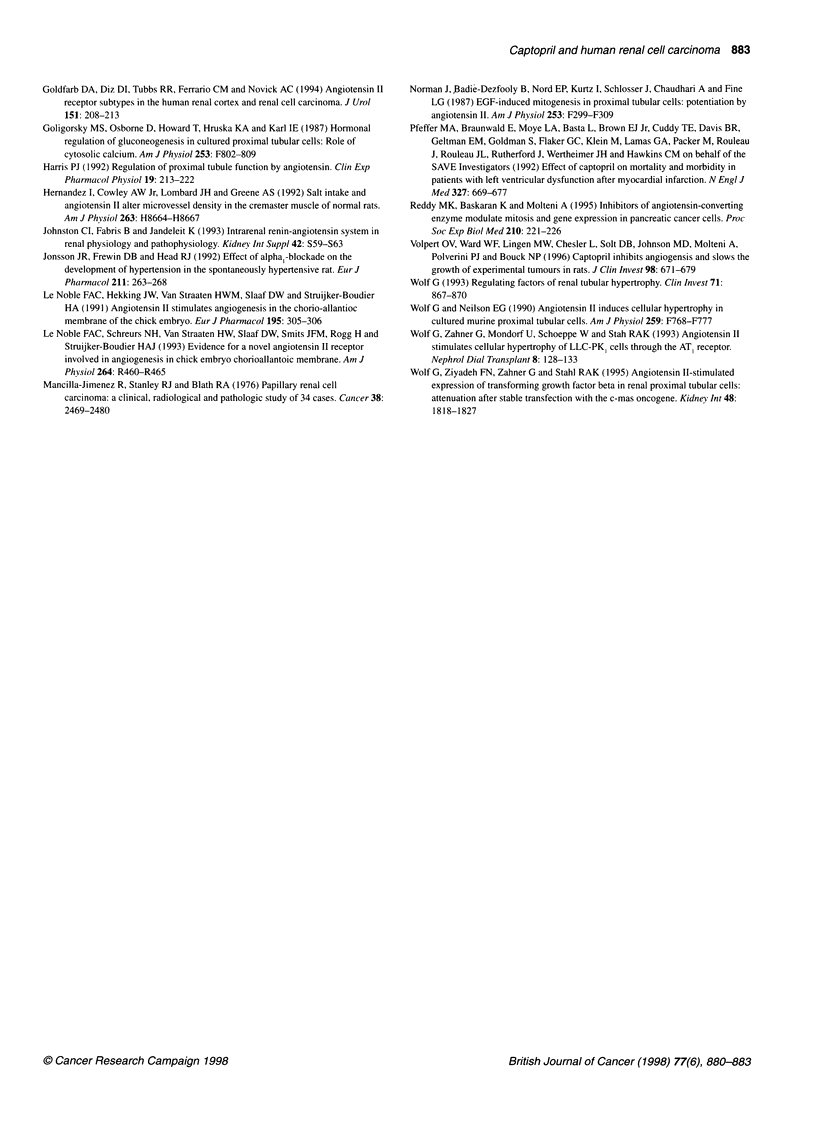# Captopril inhibits tumour growth in a xenograft model of human renal cell carcinoma.

**DOI:** 10.1038/bjc.1998.145

**Published:** 1998-03

**Authors:** S. I. Hii, D. L. Nicol, D. C. Gotley, L. C. Thompson, M. K. Green, J. R. Jonsson

**Affiliations:** Department of Surgery, University of Queensland, Princess Alexandra Hospital, Woolloongabba, Australia.

## Abstract

**Images:**


British Journal of Cancer (1998) 77(6), 880–883
© 1998 Cancer Research Campaign

### Captopril inhibits tumour growth in a xenograft model of human renal cell carcinoma

**S-I Hii, DL Nicol, DC Gotley, LC Thompson, MK Green and JR Jonsson**

Department of Surgery, University of Queensland, Princess Alexandra Hospital, Woolloongabba, Queensland 4102, Australia

**Summary** The effect of captopril on tumour growth was examined in a xenograft model of human renal cell carcinoma (RCC). Inoculation of the human RCC cell line SN12K-1 ($10^6$ cells) under the left kidney capsule of severe combined immunodeficient (SCID) mice resulted in the growth of large tumours, with an increase in weight of the inoculated kidney of 3.69 ± 1.63-fold (mean ± s.d.) when compared with the contralateral normal kidney. In mice treated with captopril (19 mg $kg^{-1}$ $day^{-1}$ or 94 mg $kg^{-1}$ $day^{-1}$ administered in the drinking water), there was a significant dose-related reduction in tumour development; the tumour bearing kidneys weighed 1.9 ± 0.42 and 1.55 ± 0.42 times the normal kidneys, respectively ($P < 0.05$ compared with untreated animals). In vitro, captopril at clinically achievable doses (0.1–10 μM) had no significant effect on the incorporation of [$^3$H]thymidine into SN12K-1 cells. Thus, this highly significant attenuation by captopril of in vivo tumour growth does not appear to be due to a direct effect on the proliferation of the tumour cells. Further studies are required to determine the mechanism of inhibition of tumour growth by captopril, in particular to evaluate the role of angiotensin II in this process.

**Keywords:** captopril; renal cell carcinoma; angiotensin; tumour; SCID

Renal cell carcinoma (RCC) is the most common malignancy arising in the adult kidney. It accounts for 2% of all malignancies as well as 2% of overall cancer deaths (Mancilla-Jimenez et al, 1976). Currently, radical nephrectomy remains the only effective means of treatment for localized RCC, whereas metastatic spread is infrequently amenable to surgery and most patients die within 1 year of diagnosis. Treatment modalities including radiotherapy, chemotherapy and immunotherapy, which have proven efficacy in other malignancies, rarely alter the natural history of RCC. Treatment of systemic disease with chemotherapy and immunotherapy are both associated with significant toxicity and morbidity.

RCCs are solid tumours arising from the proximal convoluted tubules of the kidney. Tumour growth is critically dependent on abundant neovascularization mediated by vasculogenic cytokines produced by tumour cells (Folkman and Klagsburn, 1987). As a consequence of this neovascularization alterations in renal blood flow occur with RCC.

The renin angiotensin system (RAS) regulates normal kidney blood flow and fluid homeostasis, and also plays a key role in blood pressure control (Pfeffer et al, 1992; Burris, 1995). Renin, produced by the juxtaglomerular apparatus, cleaves angiotensinogen to angiotensin I (ATI). Angiotensin-converting enzyme (ACE) results in the subsequent production of the active compound angiotensin II (ATII). Although the reaction catalysed by renin is substrate specific, a number of substrates are reported to be cleaved by ACE. These include bradykinin and substance P. The actions of ATII are mediated through cell-surface membrane receptors. Within the kidney ATII receptors are expressed in vascular, glomerular and tubular structures (Douglas, 1987). The ATII receptors are also expressed in RCC (Goldfarb et al, 1994). However, the role of ATII and its receptors in RCC has yet to be defined.

Captopril (D-3-mercapto-2-methylpropanoyl-L-proline) is an orally active inhibitor of ACE that is widely used as an antihypertensive agent. In addition to its blood pressure-lowering properties, captopril dramatically reduces the cardiac and vascular hypertrophy that accompanies prolonged hypertension, an effect not seen with other antihypertensive agents such as α-antagonists or β-blockers (Antonaccio et al, 1979; Giudicelli et al, 1981; Jonsson et al, 1992). Although chronic administration of captopril to young rats (from 4 to 12 weeks of age) results in the development of a restricted and fragile vasculature, the vascular effects of captopril when administered to adult rats are less pronounced (J Jonsson, personal observation). This would suggest that captopril may influence angiogenesis without conspicuous effects on established blood vessels.

In this study we examined the effect of captopril on the growth of human RCC in vitro and in an orthotopic xenograft model.

### MATERIALS AND METHODS

### Animals

Male severe combined immunodeficient (SCID) mice were purchased at 7 weeks of age from Animal Resources Centre, Perth, Australia. The mice were kept in a temperature-controlled, specific pathogen-free room (23°C; 12 h light–dark regime) and were given free access to the standard diet (Norco, Lismore, NSW, Australia). The experiments were started when the animals were 8 weeks of age. The study was approved by the Group 5 Animal Experimental Ethics Committee, Queensland University, Australia.

*Received 30 April 1997*
*Revised 26 August 1997*
*Accepted 3 September 1997*

*Correspondence to:* Julie Jonsson, University of Queensland, Department of Surgery, Princess Alexandra Hospital, Ipswich Road, Woolloongabba, Queensland, 4102, Australia

**Figure 1** Kidneys inoculated with SN12K-1 cells (upper) and contralateral normal kidneys (lower) from control mice (left) and mice treated with captopril (94 mg $kg^{-1}$ $day^{-1}$) (right)

**Figure 2** Relative increase in kidney weight 6 weeks after inoculation with SN12K-1 cells under the left kidney capsule in control mice and mice that received captopril (19 or 94 mg $kg^{-1}$ $day^{-1}$). $^{**}P < 0.01$; $^{***}P < 0.001$ (ANOVA)

### Xenograft model of human RCC

The human RCC cell line, SN12K-1, derived from a male Caucasian RCC patient with lung metastasis was a generous gift from Dr Isaiah Fidler, Houston, TX, USA. Cells were cultured in RPMI-1640 medium (Gibco) containing 25 mM Hepes that was supplemented with 10% fetal calf serum (FCS, Gibco), 100 U $ml^{-1}$ penicillin, 100 μg $ml^{-1}$ streptomycin (ICN) and 2 mM L-glutamine (ICN) (complete medium). Before xenografting into the mouse, SN12K-1 cells reaching confluence were trypsinized using 0.25% trypsin EDTA (Gibco) and washed twice with sterile 1 × phosphate-buffered saline (PBS), pH 7.0. Cell viability was assessed by trypan blue exclusion and was always greater than 98%.

One million viable SN12K-1 cells were inoculated subcapsularly into the left kidney of each mouse. Six weeks after inoculation, the mice were killed and examined for tumour growth. Tumour burden was assessed by comparing the weight of the kidney into which RCC cells were implanted to the animal's normal contralateral kidney.

**Figure 3(A)** Effect of captopril (0–10 mM) on the in vitro proliferation of SN12K-1 cells, as assessed by [$^3$H]thymidine incorporation. $^{**}P < 0.01$ (ANOVA). **(B)** Effect of captopril on the growth curve of SN12K-1 cells (○, control; captopril: □, 0.1 μM; ■, 1 μM; △, 10 μM; ▲, 100 μM; ◇, 1 mM; ◆, 10 mM. $^{**}P < 0.01$, $^{***}P < 0.001$ (ANOVA)

### Drug treatment

After inoculation, mice ($n$ = 6 in each group) were treated with captopril (19 mg $kg^{-1}$ $day^{-1}$ or 94 mg $kg^{-1}$ $day^{-1}$) in the drinking water for 6 weeks. Drinking water containing freshly prepared captopril was changed three times a week. No captopril was given in the control group.

### In vitro cell proliferation assays

To assess the direct effect of captopril on cell proliferation, 2 × $10^4$ viable SN12K-1 cells were cultured (96-well flat-bottomed microtitre plates) in complete medium in the presence of captopril (0–10 mM). After 48 h of culture (5% carbon dioxide, 95% humidity), 1 μCi of [$^3$H]thymidine was added to each well. Sixteen hours later the cells were harvested, and the amount of [$^3$H]thymidine incorporated was measured by scintillation counting. Six replicate wells were assessed for each captopril concentration, and the results were expressed as the mean c.p.m. of the six replicates.

To assess the affect of captopril on the growth curve of SN12K-1 cells, 2 × $10^4$ viable SN12K-1 cells were cultured in complete medium in the presence of captopril (0–10 mM) and the number of

© Cancer Research Campaign 1998

cells counted at 24, 36 and 48 h after initiation of the culture. Before counting, the cells were trypsinized using 0.25% trypsin EDTA (Gibco) and washed once with sterile 1 × PBS, pH 7.0. Cell viability was assessed by trypan blue exclusion and was greater than 98% for all captopril concentrations except 10 mM. Three replicate wells were assessed for each captopril concentration at each time point and the results expressed as the mean cell number of the three replicates.

### Statistical analyses

Data are expressed as means ± s.d. Differences between groups was assessed by analysis of variance (ANOVA), with $P < 0.05$ regarded as statistically significant.

### RESULTS

Inoculation of the left kidney of SCID mice resulted in the development of large tumours (Figure 1) with a mean increase in kidney weight 3.69 ± 1.63-fold.

In those mice administered captopril in the drinking water (19 mg $kg^{-1}$ $day^{-1}$ or 94 mg $kg^{-1}$ $day^{-1}$), tumour growth was significantly reduced when compared with control mice (Figure 1). The tumour bearing kidneys in captopril treated mice weighed only 1.9 ± 0.42 and 1.55 ± 0.42 times the normal kidneys in the 19 mg $kg^{-1}$ $day^{-1}$ or 94 mg $kg^{-1}$ $day^{-1}$ groups respectively ($P < 0.05$, ANOVA) (Figure 2). Captopril treatment had no observable detrimental side-effects.

The direct effect of captopril on the proliferation of SN12K-1 cells was assessed by [$^3$H]thymidine incorporation during in vitro culture. Captopril at clinically achievable concentrations (0.1–10 μM) had no significant effect on [$^3$H]thymidine incorporation (Figure 3A). At concentrations higher than those clinically achievable (30 μM–3 mM), captopril inhibited SN12K-1 cell proliferation by 14–31%. At 10 mM, a direct cytotoxic effect was observed that resulted in cell detachment and death within 48 h of culture initiation. In addition, clinically achievable concentrations of captopril (0.1–100 μM) did not significantly affect the growth curve of SN12K-1 cells (Figure 3B).

### DISCUSSION

In the present study, the ACE inhibitor captopril was found to inhibit tumour growth in human RCC in a SCID mouse model. Significant reduction in tumour size was observed after orthotopic implantation of human RCC in captopril-treated animals compared with controls. This would not appear to be a direct effect as proliferation of RCC cells in culture was not inhibited by clinically achievable concentrations of captopril. An indirect effect of captopril, possibly as a consequence of reduction in ATII activity, would therefore appear responsible for the reduction in tumour size.

ATII has diverse effects on the proximal tubular cells from which RCCs arise. It modifies fluid and electrolyte reabsorption (Cogan, 1990; Harris, 1992) and exerts metabolic effects including gluconeogenesis and ammonia production (Chobanian and Julin, 1987; Goligorsky et al, 1987; Johnston et al, 1993). In vitro ATII induces hypertrophy of proximal tubular cells (Wolf and Neilson, 1990; Harris, 1992; Wolf et al, 1993; Wolf, 1993) and potentiates the mitogenic effects of epidermal growth factor (Norman et al, 1987). It also induces the cellular oncogenes c-*myc*, c-*fos*, and c-N-*ras* (Wolf and Neilson, 1990), as well as growth factors such as TGF-β (Wolf et al, 1995). Therefore, captopril may have an antiproliferative effect on RCC through a reduction of ATII-stimulated production of growth factors or cytokines. Conversely, as ACE catalyses the cleavage of substrates other than ATII, the observed effect of captopril may be independent of the actions of ATII. Further studies using specific ATII-receptor antagonists in our animal model will determine whether the inhibition of tumour growth by captopril involves the RAS.

The tumour inhibitory effects of captopril may not be renal specific. Chemically-induced hepatic tumours in rats (Volpert et al, 1996) and pancreatic duct carcinoma in hamsters (Reddy et al, 1995) have also recently been shown to be dramatically reduced with captopril. This appears to be due to inhibition of mitosis as the proliferation of preneoplastic cells was reduced by captopril before actual tumour formation.

Less direct effects of ATII include vascular changes associated with hypertension and, specifically, contraction, increased protein synthesis and hypertrophy of vascular smooth muscle cells. It may also have a role in neovascularization. Recent studies have shown ATII-dependent stimulation of angiogenesis in experimental models (Fernandez et al, 1985; Le Noble et al, 1991; 1993) and enhanced vessel density in muscle tissue (Hernandez et al, 1992). Captopril has been shown previously to be an effective inhibitor of angiogenesis. In the rat, it inhibits aortic and microvascular growth and prevents induction of corneal neovascularization (Volpert et al, 1996). In culture it inhibits vascular endothelial cell migration and collagenase production, which are both critical to angiogenesis in vivo. These endothelial cell effects appear independent of ACE inhibition and are not seen with other ACE inhibitors (Volpert et al, 1996).

In conclusion, we have demonstrated growth inhibition of RCC by captopril. Future studies are required to determine if this is mediated through the renin–angiotensin system and whether it is a consequence of inhibition of cell proliferation or tumour neovascularization. Clinical studies would appear to be needed to determine whether captopril may have a role as an adjuvant to surgical treatment for RCC.

### ACKNOWLEDGEMENTS

Our sincere thanks to Bristol Myers-Squibb for providing us with the captopril. We also wish to thank Drs Andrew Clouston, Lucien Ooi and Jodie Guthrie for their help and support.

### REFERENCES

Antonaccio MJ, Rubin B, Horowitz ZP, Laffan RJ, Goldberg ME, High JP, Harris DN and Zaidi I (1979) Effects of chronic treatment with captopril (SQ14,225), an orally active inhibitor of angiotensin 1-converting enzyme in spontaneously hypertensive rats. *Jpn J Pharmacol* **29**: 285–294

Burris JF (1995) The expanding role of angiotensin converting enzyme inhibitors in the management of hypertension. *J Clin Pharmacol* **35**: 337–342

Chobanian MC and Julin CM (1991) Angiotensin II stimulates ammoniagenesis in canine renal proximal tubule segments. *Am J Physiol* **260**: F19–F26

Cogan MG (1990) Angiotensin II: a powerful controller of sodium transport in the early proximal tubule. *Hypertension* **15**: 451–458

Douglas JG (1987) Angiotensin receptor subtypes of the kidney cortex. *Am J Physiol* **253**: F1–F7

Fernandez LA, Twickler J and Mead A (1985) Neovascularisation produced by angiotensin II. *J Lab Clin Med* **105**: 141–145

Folkman J and Klagsburn M (1987) Angiogenic factors. *Science* **235**: 442–447

Giudicelli JF, Richer C, Freslon JL, Glasson S and Decourt S (1981) Comparison of the effects of beta-blockers and captopril on the development of genetic hypertension in the rat. *Arch Mal Coeur Vaiss* **74**: 51–59

© Cancer Research Campaign 1998

Goldfarb DA, Diz DI, Tubbs RR, Ferrario CM and Novick AC (1994) Angiotensin II receptor subtypes in the human renal cortex and renal cell carcinoma. *J Urol* **151**: 208–213

Goligorsky MS, Osborne D, Howard T, Hruska KA and Karl IE (1987) Hormonal regulation of gluconeogenesis in cultured proximal tubular cells: Role of cytosolic calcium. *Am J Physiol* **253**: F802–809

Harris PJ (1992) Regulation of proximal tubule function by angiotensin. *Clin Exp Pharmacol Physiol* **19**: 213–222

Hernandez I, Cowley AW Jr, Lombard JH and Greene AS (1992) Salt intake and angiotensin II alter microvessel density in the cremaster muscle of normal rats. *Am J Physiol* **263**: H8664–H8667

Johnston CI, Fabris B and Jandeleit K (1993) Intrarenal renin-angiotensin system in renal physiology and pathophysiology. *Kidney Int Suppl* **42**: S59–S63

Jonsson JR, Frewin DB and Head RJ (1992) Effect of alpha$_1$-blockade on the development of hypertension in the spontaneously hypertensive rat. *Eur J Pharmacol* **211**: 263–268

Le Noble FAC, Hekking JW, Van Straaten HWM, Slaaf DW and Struijker-Boudier HA (1991) Angiotensin II stimulates angiogenesis in the chorio-allantioc membrane of the chick embryo. *Eur J Pharmacol* **195**: 305–306

Le Noble FAC, Schreurs NH, Van Straaten HW, Slaaf DW, Smits JFM, Rogg H and Struijker-Boudier HAJ (1993) Evidence for a novel angiotensin II receptor involved in angiogenesis in chick embryo chorioallantoic membrane. *Am J Physiol* **264**: R460–R465

Mancilla-Jimenez R, Stanley RJ and Blath RA (1976) Papillary renal cell carcinoma: a clinical, radiological and pathologic study of 34 cases. *Cancer* **38**: 2469–2480

Norman J, Badiė-Dezfooly B, Nord EP, Kurtz I, Schlosser J, Chaudhari A and Fine LG (1987) EGF-induced mitogenesis in proximal tubular cells: potentiation by angiotensin II. *Am J Physiol* **253**: F299–F309

Pfeffer MA, Braunwald E, Moye LA, Basta L, Brown EJ Jr, Cuddy TE, Davis BR, Geltman EM, Goldman S, Flaker GC, Klein M, Lamas GA, Packer M, Rouleau J, Rouleau JL, Rutherford J, Wertheimer JH and Hawkins CM on behalf of the SAVE Investigators (1992) Effect of captopril on mortality and morbidity in patients with left ventricular dysfunction after myocardial infarction. *N Engl J Med* **327**: 669–677

Reddy MK, Baskaran K and Molteni A (1995) Inhibitors of angiotensin-converting enzyme modulate mitosis and gene expression in pancreatic cancer cells. *Proc Soc Exp Biol Med* **210**: 221–226

Volpert OV, Ward WF, Lingen MW, Chesler L, Solt DB, Johnson MD, Molteni A, Polverini PJ and Bouck NP (1996) Captopril inhibits angiogensis and slows the growth of experimental tumours in rats. *J Clin Invest* **98**: 671–679

Wolf G (1993) Regulating factors of renal tubular hypertrophy. *Clin Invest* **71**: 867–870

Wolf G and Neilson EG (1990) Angiotensin II induces cellular hypertrophy in cultured murine proximal tubular cells. *Am J Physiol* **259**: F768–F777

Wolf G, Zahner G, Mondorf U, Schoeppe W and Stah RAK (1993) Angiotensin II stimulates cellular hypertrophy of LLC-PK$_1$ cells through the AT$_1$ receptor. *Nephrol Dial Transplant* **8**: 128–133

Wolf G, Ziyadeh FN, Zahner G and Stahl RAK (1995) Angiotensin II-stimulated expression of transforming growth factor beta in renal proximal tubular cells: attenuation after stable transfection with the c-mas oncogene. *Kidney Int* **48**: 1818–1827

© Cancer Research Campaign 1998